# The influence of dissection on clinical anatomical knowledge for surgical needs

**DOI:** 10.1007/s00276-021-02802-w

**Published:** 2021-07-26

**Authors:** Georg Feigl, Andreas Sammer

**Affiliations:** 1 Institute of Anatomy and Clinical Morphology, Witten/Herdecke University, Alfred-Herrhausen-Straße 50, 58455 Witten, Germany; 2 Department of Macroscopic and Clinical Anatomy, Medical University of Graz, Graz, Austria

**Keywords:** Entire human body dissection, Dissection course, Education, Medical curriculum, Topographic anatomy

## Abstract

**Purpose:**

Due to the ongoing discussion of the usefulness of dissection on human bodies in medical curricula, we investigated the influence of anatomical knowledge collected in the dissection course and requested for modules of visceral surgery.

**Methods:**

Students attending the dissection course of topographic anatomy had to answer a questionnaire of 22 questions with focus on anatomical knowledge required for visceral surgical modules. Failure was defined as 13 or fewer correct answers, success categorized as high, good or moderate. The same questionnaire was handed out to 245 students prior to the module on visceral surgery. Students provided information on which regions they had dissected during the course or prior to the module. The results were compared to the result of a written Multiple Choice Question (MCQ) exam of the module visceral surgery (*n* = 160 students) with an unannounced primary focus on anatomy.

**Results:**

Students who dissected the truncal regions of the human body succeeded in answering the questionnaire with high success. Students dissecting regions of the Head/Neck or Limbs had a high failure rate, and none of them reached the “high” success level. In the MCQ exam, students dissecting truncal regions had a high success rate, while those who had not dissected or who dissected the Head/Neck or Limbs had a high failure rate.

**Conclusion:**

Dissections support and improve the required knowledge for surgical modules. For the visceral surgical module, students dissecting the region prior to the module greatly benefited. Therefore, entire human body dissection assumes to be preferable.

**Supplementary Information:**

The online version contains supplementary material available at 10.1007/s00276-021-02802-w.

## Introduction

Anatomical dissection courses are basic components in most of the medical curricula in countries with access to bodies donated to science and teaching (bdtsat). Some authors saw the dissection course as the “gold standard” in anatomical teaching [7, 16, 17]. The main aim of such courses, which regularly are performed in the first 2 years, is that students get practical access to learn basic anatomical morphology, systematic anatomy and to understand complex three-dimensional topography. Unfortunately, some countries do not have access to bdtsat or cannot meet the financial requirements as dissection courses regularly are connected with high expenses for universities [1, 18]. Therefore, universities implemented other teaching methods as alternatives, such as problem-based learning (PBL), problem-oriented learning (POL), teaching on prosections and on plastinated specimens as additional techniques, and simultaneously decreased dissection hours [5, 11–13, 20, 21]. Some even completely replaced the dissection course with one of the listed methods. Other anatomists have predicted that dissection courses would vanish from medical curricula [9]. From the surgical point of view, this would be a catastrophic scenario, because many surgeons and anatomists see the dissection course as one of the main ways to collect the required anatomical knowledge [7, 19].

However, in terms of vertical integration of anatomy in the medical curriculum [15], anatomical knowledge should not be used only for systematic or topographic anatomy, but in the clinical anatomical sense as well; it is seen as the basis for understanding surgical procedures, approaches, possible complications and pathological changes. Ramsey-Stewart et al. [22] reported the high value of whole body dissections and improvement of students’ knowledge in anatomy during and directly after the dissection course. Böckers et al. [6] documented similar results in a project called “ready for the OP”, where students gained very satisfying success rates. The question arises how the dissection course has an influence on the anatomical knowledge of the students. A surgical module might be completed 1–3 years after attending the dissection course. When participating in this module, students should not be overwhelmed by the stress of relearning anatomy, and should instead concentrate on the clinical contents of the module, which should be mainly surgical. In these modules, students routinely have their first experiences in the operation room, assisting surgeons.

In July 2013, the result of a written MCQ exam in the “general surgical module” with a focus on anatomy (half of the questions were anatomical questions; the other half were surgical questions) indicated that students who attended the dissection course with the main topic “trunk of the human body” had greater success in the exam. Students who had not attended a dissection course in this topic had a high failure rate.

Therefore, the aim of this investigation is whether and/or how the anatomical knowledge, that students collected during the dissection course, influenced the anatomical knowledge required for modules of general surgery. Students dissecting only limbs or the head and neck area and learned the topographic anatomy of the trunk theoretically might be lacking in knowledge for the surgical modules.

## Methods

Before describing the investigation, we have to present **the Status quo in Graz in the year 2013–2014:**

Basic anatomical education was integrated in the first three semesters. The most important anatomical information for the surgical module was provided in the third semester.

- First semester: osteology; including lectures about the skull and practical exercises about bones of the limbs
- Second semester:
  o Module of locomotion (M4) including lectures of general arthrology, special arthrology and myology. An obligatory dissection course with a length of 5 weeks including four oral exams during the course and a final oral exam after passing the dissection course successfully. (the oral anatomical exam is connected to physics and physiology in a parallel written short answer question exam)
  o Module of communication (M5): lectures about the central nervous system and sense organs; practical exercises regarding the peripheral nervous system with a focus on the topography of nerves of the limbs without dissection (3 h per limb). Module length of 5 weeks and written MCQ exam. (including histology and physiology)
- Third to ninth semester: parallel to the compulsory obligatory modules. Optional track of “Topographic anatomy” (or special studying module, also known as SSM): dissection course with parallel lectures. The course started mid-December and lasted until the end of January or the first week of February. Ten students dissected one bdtsat, divided into two groups of five, dissecting 5 days per week (the first group from 14:00 to 16h15; the second group from 16h30 to 19h15). To provide the dissection of the entire bdtsat, three “regions” were available.
  o Head and neck (two students bilaterally; one student per group)
  o Trunk (four students in the first group; two dissecting the thorax, two the abdominal cavity)
  o Upper and lower limbs: (four students in the second group, two dissecting the limbs bilaterally; changing from upper to lower during the course)

Parallel public lectures that were optional for all students were provided on the following topics:

- Topography of the head and neck
- Topography of the thoracic cage and abdominal cavity
- Topographic anatomy of the vessels and nerves of the limbs

Oral exams during the course:

Oral mandatory exams had to be passed by all students in the course:

- Exam on peritoneal cavity (excluding the detailed blood supply, lymph drainage and innervation)
- Thoracic cage (main focus on pleural cavity, content and pericardial cavity)
- Neck
- Final exam only on the dissected region:
  o Head and neck
  o Trunk
  o Upper and lower limbs

The dissection course can be taken for the first time in the third semester, but students might, as an option, dissect other regions in the upcoming “clinical” years parallel to the clinical modules.

Since the human body does not consist exclusively of truncal areas and the Department of Anatomy had to provide dissection of all regions of the body, third semester students were mainly allocated to either the limb or head and neck regions. Therefore, if students wanted to dissect the abdominal cavity (including the peritoneal cavity and extraperitoneal spaces on their own) students had to register for a second time. Each year, 360 human medical students and 36 dental medical students started their curriculum. Although optional, 95% of students attended this course at least twice, so students dissected other regions in later semesters. This explains why 480–560 students attended the course each year between 2004 and 2014.

“Surgical module”, also known as M17 (5 weeks in length; “Orthopedics and Traumatology” are excluded): the module starts with a repetition of physiology and anatomy on the second day. Anatomy provided lectures (four academic hours), with a focus on repetition of the abdominal cavity, including the inguinal region and posterior mediastinum. Two additional academic hours practicum are provided to repeat the anatomy of the peritoneal cavity on the bdtsat. Students might be allocated to this module in their third to fifth year, thus, it might be possible that students have never dissected the abdominal or thoracic cavity prior to attending the surgical module.

### The investigation

We assessed two groups. Group 1 (Group “Topo”): this group included students passing the optional track “Topographic anatomy”. Group 2 (Group “Surgery”): students participating the MCQ “Surgical Module” formed this group.

Group 1 (group “Topo”): students answered a questionnaire with 22 anatomical MCQs that were set by the anatomist who performed the lectures and the practical course for the surgical module. This anatomist is an experienced anatomist with more than 25 years of clinical anatomical expertise. Two general surgeons assessed the questions prior to the “investigation” and confirmed their usefulness (Attachment 1). The questionnaires were distributed to students having attended the SSM or the surgical module in the Winter Semester 2013/14. Students completed the questionnaire voluntarily and had to provide data on which SSMs they have had attended and successfully passed. All students filled out the questionnaire on an individual basis, so that no teamwork could take place.

According to the number of questions (*n* = 22), we categorized the following groups according to the number of correct answers:

1. High success rate: 22–20 correct answers
2. Good success rate: 19–16 correct answers
3. Moderate success rate: 15–14 correct answers
4. Failure: 13 or fewer correct answers (would have been less than 60% on a regular exam)

The questionnaires were collected and the answers documented. We subcategorized Group 1 (Group “Topo”) as follows: group (Group “Topo”) 1/A: students with attendance in the SSM “Trunk”. Group (Group “Topo”) 1/B: students with no attendance in the SSM “Trunk”, in which the following subgroups were defined: subgroup (Group “Topo”) 1/B1: “no SSM at all”. Subgroup (Group “Topo”) 1/B2: only SSM “Limbs”. Subgroup (Group “Topo”) 1/B3: only SSM “Head and Neck”. Subgroup (Group “Topo”) 1/B4: SSMs “Limbs” and “Head and Neck”.

In addition, the results of the questionnaires distributed were compared to the results of the MCQ exams for the surgical module (M17) in July 2013 noted above.

Group 2 (group “surgery”) or MCQ exam M17: this M17 exam, which focused mainly on Anatomy, was very revealing. Usually, only five questions on Anatomy were included out of the 60 questions in total. In this exam, without the prior knowledge of the students, the responsible academics included an equal number of questions for surgery and for anatomy (60 questions in total, 30 each for Anatomy and Surgery). Twenty anatomical questions were completely new, while 10 anatomical questions were taken from the existing pool of questions. We evaluated how students with or without SSMs performed (so categorized to the corresponding groups of Group 1 (“Topo” Group) and compared these results to the results of Group 1 (Group “Topo”).

## Results

Group 1 (group “Topo”) (***Table ******1***): about 91% of the students who attended the SSM “trunk” (Group 1/A) prior to the surgical module were successful, primarily in the category “good success”. Only 6 students failed, with 8–12 correct answers. Of the 175 students in Group 1/B, however, 162 (92.6%) failed to successfully complete the exam. Only 13 students achieved “moderate” or “good” levels of success, and no one at all achieved the “high success” level. In terms of subgroups 1/B1 to 1/B4, the failure rate was, according to the number of students allocated to each subgroup, for 1/B1 92.9%, 1/B2 91.8%, 1/B3 100% and 1/B4 88.9%. Most of the 162 students who were unsuccessful answered 2–9 (138 students: 85%) questions correctly, while 24 students (15%) were close to achieving a successful level (10–12 correct answers).

**Table 1:** Result of success rate questionnaire

Group 1 (group “Topo”) total number of students: *n = *245	Failure: 0–13	Moderate success: 14–15	Good success: 16–19	High success: 20–22
Group 1/A: SSM trunk : *n* = 70	6 (8.6%)	12 (17.1%)	36 (51.4%)	16 (22.9%)
		Positive: 64 (91.4%)		
Total result group 1/B: *N* = 175	162 (92.6%)	7 (4%)	6 (3.4%)	-
		Positive 13 (7.4%)		
1/B1: no SSM at all: *n* = 98	91 (92.9%)	3 (3.1%)	4 (4%)	-
		Positive: 7 (7.1%)		
1/B2: SSM limbs: *n* = 61	56 (91.8%)	3 (4.9%)	2 (3.3%)	-
		Positive: 5 (8.2%)		
1/B3: SSM “head and neck” *N* = 7	7 (100%)	-	-	-
		Positive: 0		
1/B4: SSM limbs and “head and neck” *N* = 9	8 (88.9%)	1 (11.1%)	-	-
		Positive: 1 (11.1%)		

Group 2 (group “surgery”), MCQ exam M17 (***Table ******2***): of the 160 students who took the exam, 39 had already participated in the SSM “trunk”, and 121 students had not taken this SSM. The total failure rate was 73 students (45.6%), of whom only 7 of 160 (9.6%) who participated in SSM “trunk” failed to succeed. 87 of 160 passed the exam (54.4%) with 32 of 160 students corresponding to Group 1/A (Group “Topo”; 20% of 160 students). The failure rate for students corresponding to Group 1/B (Group “Topo”) was 54.6%, and for Subgroup 1/B1 63.2%, Subgroup 1/B2 47 1% and Subgroup 1/B3 69.2%

**Table 2:** Result of the MCQ exam Group 2 (Group “Surgery”)

MCQ exam M17; July 2013: *n* = 160	Failure = negative mark (less than 40 correct answers)	Positive (40–60 correct answers of 60 questions)
Corresponding to group 1/A SSMs including, trunk: *n* = 39	7 of 39 (17.9%)	32 of 39 (82.1%)
Corresponding to group “1/B”: no SSM “trunk”: *n* = 121	66 of 121 (54.6%)	55 of 121 (45.4%)
Corresponding to subgroup 1/B1: No SSM: *n* = 38	24 of 38 (63.2%)	14 of 38 (26.8%)
Corresponding to subgroup 1/B2: SSM, limbs: *n* = 70	33 of 70 (47.1%)	37 of 70 (52.9%)
Corresponding to subgroup 1/B3: SSM head and neck: *n* = 13	9 of 13 (69.2%)	4 of 13 (30.8%)
Total result: *n* = 160	73 (45.6%)	87 (54.4%)

MCQ exam M17 (Table 2): information is included which SSMs the students attended or whether they attended any SSM

The MCQ exam (Group 2 or group “Surgery”) demonstrated a similar distribution between success and failure to Group 1 (Group “Topo”). Students who had attended the SSM “trunk” were mostly in the group of students with a high rate of success.

## Discussion

The study clearly demonstrates the importance of “whole body dissection” and its influence on the knowledge required for clinical modules such as General Surgery. It confirms the conclusions and results of Ramsey-Stewart et al. [22], that a “whole body dissection” improves the requisite anatomical knowledge after the dissection course. In addition, our study strengthens the general opinion that the knowledge further improves when the student dissects the region of interest required for the clinical module. Students who dissected the trunk region were able to pass the surgical exam and to answer the anatomical questions with a higher success rate than students who had dissected other regions of the body. This is in accordance with the statement of Cuddy et al. [10] that dissection creates better knowledge.

As anatomists such as Older [19] stated dramatically, the teaching of anatomy in surgical specialities must be improved, highlighting that dissection on bdtsat remains the most powerful tool. Böckers et al. also concluded that an additional elective program called “Ready for the OR” supports students during their preparation for the surgical modules [6].

Replacement of this method by cyber cadavers or a virtual dissection table is not an alternative, although it can be a very useful supplement. However, such supplemental methods are by no means worthless. A virtual dissection table is useful for repetition and can be a helpful tool in countries with no access to bdtsat. However, Collins et al. [9], Cahill et al. [7], and McLachlan et al. [18] warned that unpredictable problems caused by the decrease in dissection hours in medical curricula could amount to a sword of Damocles. In addition, the clinical preparedness of trainees could suffer negative consequences [3].

Ramsey-Stewart et al. [22] reported that the replacement of dissection courses creates a lack of anatomical knowledge and consequent disadvantages for young surgeons in their training. Their implementation of a “whole body dissection course” with a length of 7 weeks resulted in significant improvement in anatomical knowledge as shown by the difference between pre-course and post-course assessments.

Therefore, dissection does influence anatomical knowledge. Our investigation demonstrates that it is also of great importance which region is dissected by a student or a young surgeon. While this might be a logical assumption, more significantly, it is proven by the current manuscript. Students who dissected “limbs” or “head and neck” had dramatically high failure rates. These failure rates did not differ to students who have not had dissected at all. Further, the dissection system implemented in the curriculum in Graz in 2013/14 was problematic in that it created students who were well prepared in their dissected region, but showed a lack of anatomical knowledge in other regions that they had not dissected. The results of this study suggest that implementing a rotational dissection system assumed to be taken into consideration, which confirms findings of several publications [2, 4, 14, 23, 24].

We hypothesize that the result of our study was caused by an important change in the medical curriculum in Graz with the advent of the new century. Previously, the exam on Anatomy (entire human body) compensated for any “lack of knowledge” arising from shortcomings in the dissection course since students were required to study the entire anatomy to succeed in the Anatomy exam. With the change of the curriculum, the strict allocation to the dissection of regions continued, and the dissection course program remained unchanged. However, the curriculum concerning exams changed dramatically. While previously, students had to pass separate exams in histology, physiology, biochemistry, and so on, not a single exam remained. Instead, modules were implemented which included different specialities. For example, the locomotion module included anatomy, physiology and physics, but the exam itself focused only on the knowledge of locomotion. Unfortunately, the change to module exams no longer compensated for the lack of knowledge gained previously from whole body dissections, thus eliminating a very important safeguard.

So was there any advantage of the new curriculum in Graz? We believe so, yes. The advantage was that students could attend the dissection course several times and perform a whole body dissection until the end of their studies. The disadvantage of this system, however, was that a student could finish studying without having dissected at all in terms of topographic anatomy, and thus not having acquired any of that knowledge. Interestingly, the failure rate of students who had not participated in SSM did not differ from the failure rate of students passing SSMs of the “Head and Neck” or “Limbs”. Thus, the conclusion is clear that the more a student dissects actively, the better the knowledge is retained.

In summary, the manuscript clearly documents the advantage of whole body dissection and confirms the findings of Ramsey-Stewart et al. [22]. In cases of reduction of dissection hours, an exam on the entire human body could compensate for the resulting lack of knowledge. The weakest scenario would be a dissection course with allocated regions, no possible second or third dissection course and the inclusion of only modules that do not cover the anatomy of the entire human body.

A dissection of a region strengthens the anatomical basics for the clinical modules. However, it should be stated that a short repetition of the knowledge by performing lectures or practical exercises in the anatomy lab is recommended for vertical integration [15].

## Supplementary Information

Below is the link to the electronic supplementary material.

Supplementary file1 (DOCX 27 KB)

## References

[1] Aziz MA, McKenzie JC, Wilson JS, Cowie RJ, Ayeni SA, Dunn BK (2002). The human cadaver in the age of biomedical informatics. Anat Rec.

[2] Bogduk N (2001). Art macabre: is anatomy necessary?. ANZ J Surg.

[3] Bohl MA, Gest TR (2011). Resident perceptions of anatomy education: A survey of medical school alumni from two different anatomy curricula and multiple medical specialities. Anat Sci Educ.

[4] Bokey L, Chapuis P (2001). Art macabre: is anatomy necessary?. ANZ J Surg.

[5] Bouwer HE, Valter K, Webb AL (2016). Current integration of dissection in medical education in Australia and New Zealand. Anat Sci Educ.

[6] Böckers A, Lippold D, Fassnacht U, Schelzig H, Böckers TM (2011). Ready for the OR? Clinical anatomy and basic surgical skills for students in their preclinical education. GMS.

[7] Cahill DR, Leonard RJ, Marks SC (2000). Standards in health care and medical education. Clin Anat.

[8] Chapman SJ, Hakem AR, Marangoni G, Prasad KR (2013). Anatomy in medical education: perceptions of undergraduate medical students. Ann Anat.

[9] Collins TJ, Given RL, Hulsebosch CE, Miller BT (1994). Status of gross anatomy in the US and Canada. Dilemma for the 21st century. Clin Anat.

[10] Cuddy MM, Swanson DB, Drake RL, Pawlina W (2013). Changes in anatomy instruction and USMLE performance: empirical evidence on the absence of a relationship. Anat Sci Educ.

[11] Drake RL, McBride JM, Lachman N, Pawlina W (2009). Medical education in the anatomical sciences: the winds of change continue to blow. Anat Sci Educ.

[12] Drake RL (2014). A retrospective and prospective look at medical education in the United States: trends shaping anatomical sciences education. J Anat.

[13] Drake RL, McBride JM, Pawlina W (2014). An update on the status of anatomical sciences education in United States medical schools. Anat Sci Educ.

[14] Fahrer M (2001). Art macabre: is anatomy necessary?. ANZ J Surg.

[15] Fasel JH, Morel P, Gailloud P (2005). A survival strategy for anatomy. Lancet.

[16] Fillmore EP, Brokaw JJ, Kochar K, Nalin PM (2016). Understanding the current anatomical competence landscape: comparing perceptions of program directors, residents, and fourth-year medical students. Anat Sci Educ.

[17] Kerby J, Shukur ZN, Shaloub J (2011). The relationships between learning outcomes and methods of teaching anatomy as perceived by medical students. Clin Anat.

[18] McLachlan JC, Bligh J, Bradley P, Searle J (2004). Teaching anatomy without cadavers. Med Educ.

[19] Older J (2004). Anatomy: a must for teaching the next generation. Surgeon.

[20] Ovensek N (2013). College of medicine gross anatomy report.

[21] Pais D, Casal D, Mascarenhas-Lemos L, Barata P, Moxham BJ, Goryi-O´Neill  J (2017). Outcomes and satisfaction of two optional cadaveric dissection courses: a 3-year prospective study. Anat Sci Educ.

[22] Ramsey-Stewart G, Burgess AW, Hill DA (2010). Back to the future: teaching anatomy by whole-body dissection. MJA.

[23] Taylor TKF (2001). Art macabre: is anatomy necessary?. ANZ J Surg.

[24] Winkelmann A (2007). Anatomical dissection as a teaching method in medical school: a review of the evidence. Med Educ.

