# Supplementary material for: The influence of dissection on clinical anatomical knowledge for surgical needs
Source: Surg Radiol Anat. 2021 Jul 26;44(1):9–14. doi: 10.1007/s00276-021-02802-w (PMC8758640; doi:10.1007/s00276-021-02802-w)
Supplement: Supplementary file 1 — Supplementary file1 (DOCX 27 KB) [file 276_2021_2802_MOESM1_ESM.docx]

Attachment 1: questionnaire for Group 1 („Topo“ Group) with the englisch translation of the questions.

- Der Anulus inguinalis superficialis wird durch die Aufspaltung der Aponeurose welchen Muskels gebildet? (The superficial inguinal ring is formed by the splitting of the aponseurosis of which muscle?)

1. M. obliquus abdominis internus
2. M. obliquus abdominis externus
3. M. transversus abdominis
4. M. rectus abdominis
5. M. pectineus

- Welche Struktur begrenzt den Anulus inguinialis profundus medial? (Which structure is the medial border of the inner inguinal ring)

1. Plica umblicalis mediana
2. Plica umbilicalis medialis
3. Plica epigastrica
4. Plica gastropancreatica
5. Plica duodenalis superior

- Welche Struktur liegt in der Plica gastropancreatica? (Which structure is to be found in the gastropancreatic fold?)

1. A. gastrica dextra
2. A. gastrica sinistra
3. A. gastrica brevis
4. A. gastrica dorsalis
5. A. lienalis /splenica

- Auf welchen Wirbel projiziert sich der Hiatus oesophageus? (On which vertebral body level is the projection of the oesophageal hiatus?)

1. L2
2. Th 12
3. Th 10
4. Th 8
5. Th 6

- Welche Struktur begrenzt mir bogenartig den Hiatus aorticus? (Which arched structure forms the artic hiatus?)

1. Lig. arcuatum mediale
2. Lig. arcuatum laterale
3. Lig. arcuatum medianum
4. Psoasarkade
5. Quadratusarkade

- Welche Äste zählen zu den unpaaren Ästen der Aorta? (Which vessels are unpaired branches of the abdominal aorta?)

1. Truncus coeliacus
2. A. mesenterica superior
3. A. mesenterica inferior
4. Truncus lumbalis
5. 1, 2 und 3 richtig
6. 1 und 3 richtig
7. 2 und 4 richtig
8. Nur 4 richtig
9. Alle richtig

- Welche Aussage hinsichtlich der Aa. renales ist richtig? (Which answer concerning the renal arteries is correct?)

1. Die A. renalis dextra kreuzt vor der V. cava inferior (The right renal artery passes ventral to the inferior caval vein)
2. Die Aa. renales gehen kaudal des Abgangs der A. mesenterica inferior ab (The renal arteries originate caudal to the origin of the inferior mesenteric artery)
3. Die A. renalis sinistra liegt stets vor der V. renalis sinistra (The left renal artery always is located ventral to the left renal vein)
4. Die Aa. renales geben eine A. suprarenalis inferior ab (The renal arteries give off an inferior suprarenal artery)
5. Die A. renalis versorgt die Milz (The renal artery supplies the spleen)

- Die Bifurcatio aortae projiziert sich wohin? (On which vertebral body level or structure is the projection of the bifurcation of abdominal aorta?)

1. L1
2. Umbilicus
3. S2
4. L4
5. 1, 2 und 3 richtig
6. 1 und 3 richtig
7. 2 und 4 richtig
8. Nur 4 richtig
9. Alle richtig

- Die direkten Äste des Truncus coeliacus sind? (Which regularly are the main branches of the coeliac trunk)

1. A. gastrica sinistra
2. A. hepatica communis
3. A. lienalis (splenica)
4. A. gastroduodenalis
5. 1, 2 und 3 richtig
6. 1 und 3 richtig
7. 2 und 4 richtig
8. Nur 4 richtig
9. Alle richtig

- Die V. mesenterica superior und V. lienalis (splenica) bilden durch ihren Zusammenfluss welche Vene? (Which vein is formed by the confluens of superior mesenteric and splenic vein?)

1. V. mesenterica inferior
2. V. cava inferior
3. V. portae
4. V. hepatica
5. V. azygos

- Welche Venen werden erweitert, damit es bei portalen Hypertonus zur Ausbildung des Caput medusae kommt? (Which veins are enlarged to form the „Caput medusae “caused by portal hypertonia?)

1. V. umbilicalis
2. Venae paraumbilicales
3. V. cava inferior
4. Vv. heapticae
5. Vv. epigastricae superficiales

- Welche Gefäße laufen entlang der Curvatura maior ventriculi? (Which vessels are regularly to be found along the greater curvature?)

1. A. gastroepiploica (gastroomentalis) dextra
2. A. gastroduodenalis
3. A. gastroepiploica (gastroomentalis) sinistra
4. A. lienalis
5. 1, 2 und 3 richtig
6. 1 und 3 richtig
7. 2 und 4 richtig
8. Nur 4 richtig
9. Alle richtig

- An welcher Stelle der Leber treten die Venae hepaticae aus? (At which area of the liver do exit the hepatic veins?)

1. Porta hepatis
2. Lamina affixa
3. Fissura ligamenti venosi
4. Area nuda
5. 1, 2 und 3 richtig
6. 1 und 3 richtig
7. 2 und 4 richtig
8. Nur 4 richtig
9. Alle richtig

- Welche lymphatischen Stämme vereinen sich zur Cisterna chyli? (Which main lymphatic trunks drain into the chyle cistern?)

1. Truncus lumbalis dexter
2. Truncus intestinalis
3. Truncus lumbalis sinister
4. Ductus lymphaticus dexter
5. 1, 2 und 3 richtig
6. 1 und 3 richtig
7. 2 und 4 richtig
8. Nur D richtig
9. Alle richtig

- In welche Reservetasche der Pleura legt sich der Margo anterior der Lunge? (Which structure of the parietal pleura is used by the anterior margin of the lung during inspiration?)

1. Recessus costomediastinalis
2. Recessus costodiaphragmaticus
3. Recessus mediastinovertebralis
4. Recessus diaphragmaticomediastinalis
5. Cupula pleurae

- Welche Aussage zum Ösophagus ist falsch? (Which statement according the oesophagus is WRONG!)

  A) Er wird vom Nervus vagus begleitet (He is accompanied by the vagus nerve)
  B) Er wird kaudalwärts durch die Aorta von der Wirbelsäule verdrängt (He is pushed ventrally in the caudal posterior mediastinum by the aorta)
  C) Er wird vom Nervus phrenicus motorisch innerviert (Nerve supply os provided by the phrenic nerve)
  D) Er liegt im hinteren Mediastinum (He is located in the posterior mediastimun)
  E) Das venöse Blut aus dem Plexus oesophageus wird einerseits in den Pfortaderkreislauf und andererseits in das Hohlvenensystem abgeleitet (Venous draining of the oesphageal veins is provided into the portal system as well into the caval system)
- Welche Pleurastruktur legt spannt sich im Bereich der Apertura thoracis superior aus? (Which pleural structure is to be found in the upper thoracic opening?)

1. Pleura costalis
2. Pleura mediastinalis
3. Pleura diaphragmatica
4. Cupula pleurae
5. Lig. pulmonale

- Welche der folgenden Aussage/n zum Lungenstiel ist /sind richtig? (Which statement according to the root of lung is CORRECT?)

1. Die V. azygos zieht von caudal nach cranial hinter dem Lungenstiel vorbei (The azygos vein passest h root oft he lung dorsally in caudocranial direction)
2. Der N. phrenicus zieht hinten vorbei (The phrenic nerve passes dorsally)
3. Die A. pulmonalis verläuft im Lungenstiel (The pulmonary artery is a strucutre in the root of the lung)
4. Der N. vagus zieht vorne vorbei (The vagus nerve passes ventrally)
5. 1, 2 und 3 richtig
6. 1 und 3 richtig
7. 2 und 4 richtig
8. Nur 4 richtig
9. Alle richtig

- Welche der folgenden Aussagen sind richtig? (Which statements are CORRECT?)

1. Die Vena cava superior entsteht durch den Zusammenfluss der V. subclavia und der V. jugularis interna und mündet in das Atrium dextrum. (The superior vena cava is fromed by the confluens of the subclavian vein an internal juigular vein and drains in the right atrium)
2. Die Vena cava inferior mündet rasch nach Durchtritt durch das Zwerchfell in das Atrium sinistrum. (The inferior vena cava drains shortly after piercing the diaphragm in the left atrium)
3. Die V. cava inferior nimmt kurz vor ihrem Durchtritt durch das Foramen v. cavae die V. portae auf. (Shortly before passing the caval opening the portal vein drains into the inferior vena cava)
4. Der Truncus pulmonalis entspringt aus dem Ventriculus dexter und ist von diesem durch die Valva mitralis getrennt. (In between the reight ventricle and the pulmonoary trunk one can find the mitral valve)
5. 1, 2 und 3 richtig
6. 1 und 3 richtig
7. 2 und 4 richtig
8. Nur 4 richtig
9. Alle sind falsch

- Zwischen welchen Gefäßen gelangt man transperikaridal zur A. pulmonalis dextra? (In between which vessels you can reach the right pulmonary artery transpericardially?)

1. Aorta ascendens
2. V. cava inferior
3. V. cava superior
4. Aorta descendens
5. 1 , 2 und 3 richtig
6. 1 und 3 richtig
7. 2 und 4 richtig
8. Nur 4 richtig
9. Alle richtig

- Welche Aussage zur rechten Lunge ist richtig? (Which statement according to the right lung is CORRECT?)

1. Die Fissura obliqua projiziert sich auf die 4. Rippe (The oblique fissure projects along the 4^th^ rib)
2. Die Fissura horizontalis trennt den Lobus superior vom Lobus inferior (the horizontal fissure is in between upper and lower lobe)
3. Der Margo inferior trennt die Facies costalis von der Facies mediastinalis (The inferior margin is in between the costal and mediastinal surface)
4. Die Pleura pulmonalis ist Teil der Pleura parietalis (The pulmonary pleura is part of the parietal pleura)
5. Das Gekröse der Lunge heisst Lig.pulmonale (The “meso” of the lung is called „pulonary ligament)

- Welche Reihenfolge der Gebilde des rechten Lungenstiels von kranial nach kaudal aufgezählt ist richtig? (Which is the correct order of structures in the right root of lung from cranial to caudal?)

  A) A. pulmonalis dextra, Bronchus principalis dexter, V. pulmonalis dextra superior, V. pulmonalis dextra inferior
  B) Bronchus principalis dexter, V. pulmonalis dextra superior, A. pulmonalis dextra, V. pulmonalis dextra inferior
  C) Bronchus principalis dexter, A. pulmonalis dextra, V. pulmonalis dextra superior, V. pulmonalis dextra inferior
  D) V. pulmonalis dextra superior, Bronchus principalis dexter, A. pulmonalis dextra, V. pulmonalis dextra inferior
  E) Bronchus principalis dexter, V. pulmonalis dextra superior, V. pulmonalis dextra inferior, A. pulmonalis dextra
